# A Case of Autoimmune Hemolytic Anemia Following COVID-19 Messenger Ribonucleic Acid Vaccination

**DOI:** 10.7759/cureus.15035

**Published:** 2021-05-15

**Authors:** Sérgio Brito, Nuno Ferreira, Sofia Mateus, Manuela Bernardo, Beatriz Pinto, Ana Lourenço, Fátima Grenho

**Affiliations:** 1 Internal Medicine, Hospital CUF Tejo, Lisbon, PRT; 2 Hematology and Oncology, Hospital CUF Tejo, Lisbon, PRT; 3 Pharmacy, Hospital CUF Tejo, Lisbon, PRT

**Keywords:** covid-19 mrna vaccine, autoimmune hemolytic anemia, sars-cov-2, warm autoimmune hemolytic anemia, immune-mediated hemolysis

## Abstract

Autoimmune hemolytic anemia (AIHA) is a condition characterized by the increased destruction of red blood cells (RBCs) mediated by anti-erythrocyte autoantibodies with or without complement activation. Its clinical presentation is heterogeneous, ranging from asymptomatic to severe forms with fatal outcomes, and it can be either idiopathic or secondary to a coexisting disorder. In this report, we present a case of a patient who suffered from acute and severe AIHA after receiving the second dose of the coronavirus disease 2019 (COVID-19) messenger ribonucleic acid (mRNA) vaccine.

## Introduction

Severe acute respiratory syndrome coronavirus 2 (SARS-CoV-2) is the agent responsible for the coronavirus disease 2019 (COVID-19) pandemic [1]. Vaccination is the most effective and safest way to reduce fatalities and prevent severe illness related to COVID-19 [1,2]. In light of this, throughout the year 2020, pharmaceutical companies were engaged in the task of investigating and testing vaccines against this agent, which had led to a large-scale vaccination strategy in late 2020. All vaccines carry side effects that need to be reported and investigated in order to balance the risks and expected benefits and enable the concerned authorities to act promptly when they occur [2].

Autoimmune hemolytic anemia (AIHA) is a rare entity that is characterized by the increased destruction of red blood cells (RBCs) by anti-erythrocyte autoantibodies [Immunoglobulin G (IgG) or IgM] with or without complement activation [3]. While autoantibody-induced hemolysis occurs primarily at the extravascular level (spleen and lymphoid organs), complement-mediated lysis is mainly intravascular and intrahepatic. AIHAs can be classified as warm (wAIHA), cold (cold agglutinin disease), or mixed based on the optimal temperature of the reaction with autologous erythrocytes. The clinical presentation is heterogeneous, ranging from compensated forms without anemia to severe disease. With respect to its etiology, it can be idiopathic (without coexisting disorders) or secondary to infections, neoplasia, lymphoproliferative disorders, systemic autoimmune diseases, or drugs [3,4].

We have complied with the local regulations on reporting side effects as per the guidelines of Portugal's national regulatory authority, the National Authority of Medicines and Health Products (INFARMED) [5].

## Case presentation

An 88-year-old Caucasian woman was admitted to the emergency room (ER) with a sudden onset of asthenia and jaundice two days after receiving the second dose of the COVID-19 messenger ribonucleic acid (mRNA) vaccine (nucleoside-modified). Her medical history included insomnia and she had been taking 2 mg of estazolam at night. She denied other symptoms or any other medications and reported that she had not experienced any similar episodes in the past. The patient had received the first dose of the vaccine three weeks before without any complications. She denied any previous SARS-CoV-2 infection.

**Figure 1:**
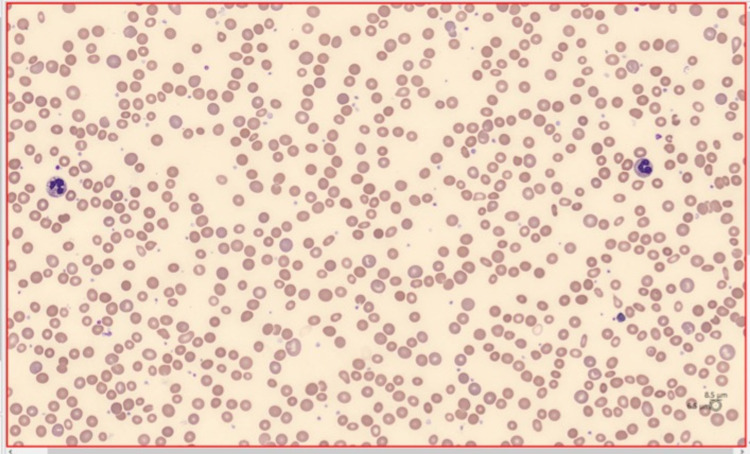
Peripheral blood smear (May-Grunwald-Giemsa staining) with normocytic and normochromic anemia, moderate anisopoikilocytosis, and moderate polychromasia

On admission, she was hemodynamically stable with jaundiced skin and mucous membranes. Blood tests revealed normocytic normochromic anemia, which was confirmed by a peripheral blood smear (Figure 1), with hemoglobin of 4.5 g/dL [reference range (r): 12-15 g/dL], hematocrit of 13.8% (r: 36-46%), mean globular volume of 89 fL (r: 80-97 fL), mean globular hemoglobin concentration of 32.6 g/dL (r: 32-36 g/dL), and reticulocytes of 0.9% (r: 0.5-1.5%), without other major morphological changes; leukogram and platelet counts were normal. Her lactate dehydrogenase (LDH) level was 1,309 U/L (r: 81-234 U/L), total bilirubin was 8.20 mg/dL (r: 0.2-1.0 mg/dL) with indirect bilirubin of 5.2 mg/dL (r: 0.2-0.8 mg/dL). Additionally, her aspartate aminotransferase (AST) level was 118 U/L (r: 15-37 U/L), alanine aminotransferase (ALT) was 37 U/L (r: 14-59 U/L), gamma-glutamyl-transpeptidase (GGT) was 25 U/L (r: 5-55 U/L), and haptoglobin was <0.3 g/L (r: 0.32-1.97 g/L). Also, she had acute kidney injury with a creatinine of 2.20 mg/dL (r: 0.55-1.02 mg/dL) and urea of 173 mg/dL (r: <50 mg/dL), with mild compensated metabolic acidosis without ionic changes. The urinalysis summary showed hemoglobinuria without bilirubinuria. The test for the SARS-CoV-2 virus using direct real-time reverse transcriptase-polymerase chain reaction (RT-PCR) assay was negative. Thoracic, abdominal, and pelvic CT showed a spleen at the upper limit of normal (13 cm), without hepatic or biliary changes suggestive of obstruction and without evidence of adenopathies or other lesions suggestive of neoplastic involvement. The direct agglutinin testing (DAT) revealed very high titers of anti-erythrocyte IgG autoantibodies and high anti-C3d titers. No cold agglutinins were detected.

**Figure 2:**
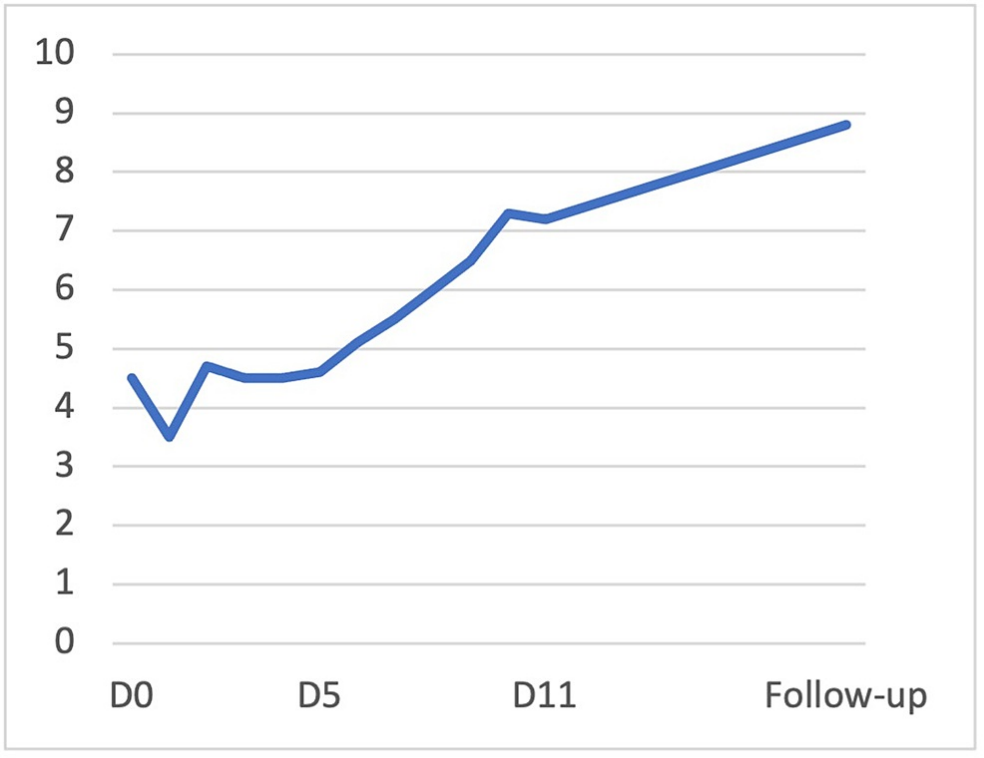
Evolution of hemoglobin values during hospitalization (g/dL)

**Figure 3:**
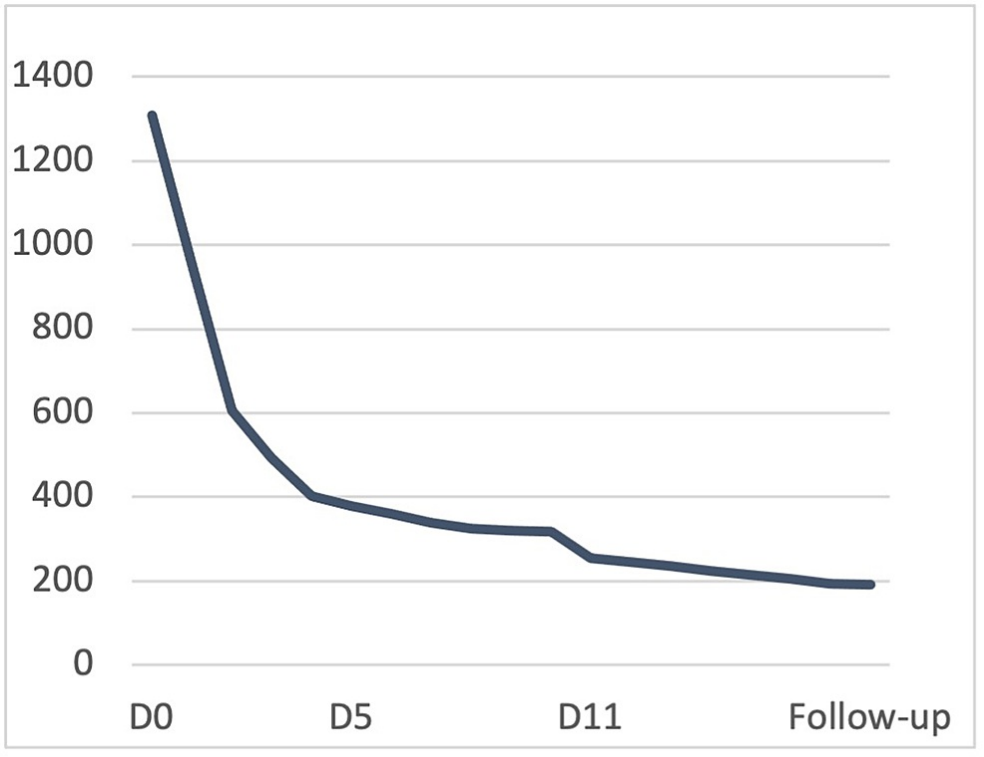
Evolution of LDH values during hospitalization (U/L) LDH: lactate dehydrogenase

In light of the above findings, a diagnosis of AIHA was assumed. The patient was admitted and therapy with methylprednisolone 1,000 mg once daily was introduced. Four hours after the admission, the patient experienced worsening fatigue and the hemoglobin dropped to 3.5 g/dL; hence, she was transfused with an erythrocyte concentrate unit with universal compatibility. She completed five days of methylprednisolone therapy and subsequent blood tests revealed stable hemoglobin levels (Figure 2), decreased LDH (Figure 3), and decreased bilirubin (<1 mg/dL after three days). On the fifth day, her counts showed reticulosis (9.3%). Her acute kidney injury showed improvement without any need for renal function replacement therapy after eight days.

During her hospitalization, active infectious diseases (human immunodeficiency virus, cytomegalovirus, Epstein-Barr virus, hepatitis A, B and C, syphilis, and *Mycoplasma pneumoniae*), as well as other concomitant autoimmune diseases (rheumatoid negative factor; normal complement factors and absence of cryoglobulins, antinuclear, anti-double-stranded DNA, and anti-cyclic citrullinated peptides antibodies), were ruled out. Immunofixation electrophoresis, quantification of immunoglobulin subpopulations, and kappa and lambda free light chains were normal. The SARS-CoV-2 test was repeated after five days of hospitalization and it remained negative.

From the sixth day of hospitalization onwards, she began to show sustained improvements in hemoglobin and hematocrit levels. The corticosteroid therapy was switched to oral with 1 mg/kg of prednisolone. On the 11th day of hospitalization, the patient was found to be asymptomatic with a hemoglobin of 7.2 g/dL. She was discharged on oral steroids and referred to internal medicine consultation. She was reevaluated a week later and found to be sustaining a rise in hemoglobin levels.

## Discussion

We presented a case of severe anemia secondary to acute intravascular and extravascular hemolysis with recovery after corticosteroid therapy. After secondary causes frequently related to the AIHA, SARS-CoV-2 infection, and other factors were excluded, and given the temporal relationship between vaccine administration and the presentation of symptoms (two days after the second dose), we hypothesized that the AIHA was triggered by the COVID-19 mRNA vaccine. The suspected side effect of the mRNA vaccine has been reported to the regulatory authority according to its guidelines.

Since the identification of the SARS-CoV-2 virus as the causative factor behind the global COVID-19 pandemic, some cases of AIHA related to acute infections have been described with or without other underlying neoplastic, lymphoproliferative, autoimmune, or infectious diseases [6]. After analyzing the studies about vaccines already approved, we found that there was a case in phase II in the control group (who received a meningococcal conjugate vaccine) [7]. We found five cases of AIHA reported by pharmaceutical companies in the weekly updated adverse reaction reports, but those have not yet been published in scientific journals [2].

AIHA had previously been described as an adverse reaction to other viral vaccines such as the influenza vaccine. Although the mechanism is not fully understood, one theory proposes an autoimmune response by molecular mimicry of host antigens by viral-derived peptides that cause cross-activation of autoreactive T or B cells [8]. Since the presentation of the condition occurred two days after receiving the second vaccination, it presupposes a secondary immune response.

Corticosteroids remain the first-line therapy for patients with secondary AIHA when you are unable to treat the underlying cause [4]. In this patient, we used high doses of corticosteroids (1,000 mg of methylprednisolone once daily for five days) followed by oral corticosteroids with suppression of hemolysis and recovery of hemoglobin and hematocrit levels.

## Conclusions

We discussed a case of a patient with severe AIHA that was successfully treated with corticosteroid therapy. After secondary causes frequently related to the AIHA were excluded and given the temporal relationship between vaccine administration and the emergence of symptoms, we hypothesized that the COVID-19 mRNA vaccine could have been the trigger for the patient's AIHA. With large-scale COVID-19 vaccination programs now being carried out on a regular basis, physicians must be alert to and quickly identify and treat possible adverse reactions and other associated complications.
